# Multidimensional associative factors for improvement in pain, function, and working capacity after rehabilitation of whiplash associated disorder: a prognostic, prospective outcome study

**DOI:** 10.1186/1471-2474-15-130

**Published:** 2014-04-16

**Authors:** Felix Angst, Andreas R Gantenbein, Susanne Lehmann, Françoise Gysi-Klaus, André Aeschlimann, Beat A Michel, Frank Hegemann

**Affiliations:** 1 Rehabilitation Clinic “RehaClinic”, Bad Zurzach, Switzerland; 2 Department of Rheumatology, Physical Medicine and Rehabilitation, University Hospital of Zurich, Zurich, Switzerland

**Keywords:** Whiplash, Prediction, Pain, Function, Coping, Catastrophizing

## Abstract

**Background:**

Whiplash associated disorders (WAD) have dramatic consequences for individual and public health. Risk factors for better and worse outcomes are important to optimize management. This study aimed to determine short- and mid-term associative co-factors of neck pain relief, improved physical functioning, and improved working capacity (dependent variables) in patients suffering from whiplash associated disorder who participated in a standardized, inpatient pain management program.

**Methods:**

Naturalistic, observational, prospective cohort study. Outcome was measured by standardized assessment instruments. Co-factors covered sociodemographics, comorbidities, social participation, affective health, and coping abilities. Stepwise, multivariate linear regression analysis was performed at discharge and at the 6-month follow-up.

**Results:**

All regression models explained high proportions of variance (53.3% – 72.1%). The corresponding baseline level was significantly associated with a change in every dependent variable (explained variances: 11.4%-56.7%). Pain relief significantly depended on improved function and vice-versa (3.4%-14.8%). Improved ability to decrease pain was associated with pain relief at discharge (9.6%). Functional improvement was associated with decreased catastrophizing (19.4%) at discharge and decreased depression (20.5%) at the 6 month follow-up.

**Conclusions:**

Pain relief, improved physical function and working capacity were associated with each other. Improved coping (catastrophizing and ability to decrease pain) and reduced depression may act as important predictors for pain relief and improved function. These findings offer toe-holds for optimized therapy of chronic WAD.

## Background

Whiplash associated disorder (WAD) and chronic neck pain after car accidents are multi-component phenomena associated with injury, physical dysfunction and maladaptive coping behavior leading to very high costs for the individual and for public health organizations [1-5]. Every year 10,000 new cases of WAD are reported in Switzerland corresponding to an annual incidence of 130/100,000 [2,3]. WAD consumes up to 50% of the costs paid by third party insurances for car accidents, i.e. up to 300 million Euro per year in Switzerland [2,3].

In the acute phase, local neck pain is associated with headache, reduced neck mobility and/or other non-specific neurological symptoms without detectable structural damage (Quebec Task Force–QTF Grade 1 and 2). In about half the cases, symptoms gradually disappear, whereas the other half may develop chronic pain syndrome within the first year post-trauma and require treatment in ambulatory or inpatient health care [2-4]. WAD is characterized by loss of physical and psycho-social function, loss of working capacity, maladaptive pain-behavior, increased activation of the autonomic nervous system expressed as hyperhidrosis, sleep disturbance, tremor, dizziness or increased muscle tone. Use of analgesics may lead to abuse with or without reduced ability to alleviate pain. Psychosocial factors may aggravate benign somatic symptoms to a severe and disabling chronic pain syndrome [5]. These factors have an effect on pain itself by exerting an influence on descending pain modulation in the prefrontal cortex, on peripheral cytokines and on anti-nociceptive mechanisms in the dorsal horn of the spinal cord [6]. Altered cognitive behavior like catastrophizing, fear of pain and avoidance strategies, expressed as ‘fear-avoidance’, may modulate the effects of post-traumatic stress on pain [7].

Psychosocial abilities, and also mental health, are considered important requisites for a better outcome [5,8]. In the most recent meta-analysis, catastrophizing but not depression was an important risk factor for chronification of WAD [9]. Psychological factors have been found to be more relevant than collision severity in predicting duration and severity of symptoms in WAD [10]. Helplessness, older age, and poor pre-injury work status were found to predict poorer health and non-recovery [9,11]. A combination of these symptoms might be triggered by whiplash injury but they might also be a manifestation of a post-traumatic or depressive disorder [12].

In a first study, state and change in bio-psycho-social health and in quality of life dimensions were examined after four weeks of interdisciplinary inpatient rehabilitation [13]. Patients showed moderate to large mid-term improvements such as reduced pain, lower intakes of analgesics, better work capacity, and improved health dimensions in health-related quality of life, anxiety and depression.

The objective of this study was to determine factors predicting pain relief, improved physical functioning, and improved working capacity in the same setting. Special focus was laid on predictors of psycho-social health and pain coping dimensions. We simultaneously examined socio-demographic and disease-relevant factors, especially age, body mass index, number of comorbidites, and the baseline score of the predictor as confounders to control for them. The present setting provided comprehensive, as well as specific assessment of health and quality-of-life, covering a large number of co-factors and assessment constructs. Existing literature does not cover the sum of these characteristics, especially with a focus on coping and inpatient rehabilitation. Therefore, the present study aims to substantially enhance current knowledge. Our main hypothesis was that psychological and pain coping factors would be strongly associated with improvements in pain and function.

## Methods

### Patients

Patients were admitted to an inpatient, interdisciplinary neck pain management program called ‘ZIHKo (Zurzacher Interdisziplinäres HWS Konzept - Zurzach Interdisciplinary Cervical Spine Concept)’ as described in a previous paper [13]. The candidates were screened in three steps prior to the pain management program: 1) Chart and diagnostic screening for fulfillment of the inclusion criteria (see below) using the admission report, 2) interview by telephone to explain the program and to assess ability and motivation to participate, 3) individual consultation to confirm previous information.

Inclusion criteria were accident 3 months to 3 years before admission, chronic neck pain for at least 3 months, age between 17 and 65 years, ‘motivation’ and agreement to participate in the therapies, and to fix ‘realistic’ goals. Exclusion criteria were: severe somatic or psychiatric comorbidities preventing participation in the program [13]. Informed consent for participation in the study was obtained from all participants. The study protocol was approved by the local ethics committee (Health Department Aarau, Switzerland, EK AG 26/2008).

### Intervention

The inpatient interdisciplinary program of 4 weeks consisted of: physiotherapy individually and in small groups, medical training therapy (MTT, graded exercise), passive therapy modules, occupational therapy, creative therapy, neuropsychological treatment with group information about pain, individualized cognitive behavioral therapy and a test psychological setting. A detailed description can be found in a previous report [13].

### Measures

Sociodemographic data and medication were collected, including a specific questionnaire and information from medical records. Comprehensive bodily pain, physical functioning, and social functioning was measured using the Short Form 36 (SF-36) [14,15]. The SF-36 is the best tested and most used generic self-report measure worldwide. The scores were compared to German general population norm scores (n = 6948), stratified by sex, age (5-year age groups), and comorbidities (present/absent) [16]. The Hospital Anxiety and Depression Scale (HADS) [17,18] was used for self-assessed affective health. Sex-and age-specific norms (10-year intervals) are available from a German population survey (n = 2037) [19]. Germany and Switzerland have the same language and cultural area. The North American Spine Society (NASS) questionnaire measures cervical spine-specific pain, (physical) function, and neurogenic symptoms [20,21]. Neck pain is assessed by two items (‘suffering from pain’ and ‘disabled by pain’), functional limitation due to neck pain by eight items. Population-based data are not available to the best of our knowledge. To assess coping in terms of cognitive and behavioral strategies of pain tolerance and pain management we used the following five scales of the Coping Strategies Questionnaire (CSQ): increasing activity, praying and hoping, catastrophizing, ability to control pain, and ability to decrease pain because these were found to be the most responsive coping scales in our previous outcome study [22-24].

### Analysis

Data were prospectively assessed on admission (baseline), at discharge (self-assessment in the clinic at both time points), and 6 months after admission (questionnaires by postal mail). Scores, score changes, and the corresponding effect sizes (score changes divided by the baseline standard deviation) were determined. To be comparable to each other all scores were scaled from 0 (worst health, most pain, no function) to 100 (best health, no pain, maximal function) as is the original scaling of the SF-36. Working capacity (hours/week) before and after the accident was obtained retrospectively from the medical records, and by asking the patients directly at baseline and at both follow-ups.

Dependent variables were change of pain (0-100 score points), change of function (0-100 score points), and change of working capacity (0-50 hours/week) between baseline and follow-up. A positive change reflected improvement, a negative worsening. All dependent variables were used as approximately continuous parameters in the multivariate linear regression models. All analyses were performed using the statistical software package SPSS 20.0 for windows (SPSS Inc., Chicago, IL, USA).

Potential associative co-factors were examined by multivariate stepwise regression models to predict the dependent variable (change in pain, function, and working capacity). Independent co-variates were: sex, age, education, partnership, number of comorbidities, body mass index (BMI), sports (hours per week), smoking, medication with analgesics or medication with antidepressants, type of accident, disease duration, CSQ activity level as potential confounders to control for them in the regression analysis. As further independent co-variates SF-36 social functioning, HADS depression, HADS anxiety, CSQ control pain, CSQ decrease pain, CSQ catastrophizing (baseline and change at follow-up for each of the scores) were included as predictors to test the hypothesis.

For the models of change of function and change of working capacity, baseline pain and change of pain were included as additional independent variables. For those of pain and working capacity, baseline function and change of function were included and for those of pain and function, baseline working capacity and change of working capacity were additionally included. For change of pain and change of function, the model that explained higher variance was presented using either the SF-36 or the NASS.

All covariates were tested by stepwise inclusion into or exclusion from the model to maximize the explained variance of the dependent variable by the model (R-squared test). The model without the covariate was compared to the model with the covariate by the F-test. Each covariate that showed a bivariate correlation to the dependent variable with a significance of p < 0.300 was included in the model: the one with the highest bivariate correlation as first, that with the second highest bivariate correlation as second etc. (stepwise inclusion). If the multivariate correlation of that variable had a significance of p < 0.100 (by the F-test) it was included in the final model (Tables 1 and 2).

**Table 1:** Multivariate regression of change in pain, function, and working capacity at discharge (n = 175)

			**Bivariate**	**Multivariate**				
**Covariate**			**Correlation**	**Partial correlation**	**Explained variance**	**F change**	**Regression coefficient**	**Coefficient’s significance**
**Pain relief: SF-36 bodily pain** (explained variance: 54.6%)								
Constant							-11.974	0.008
SF-36	Bodily pain	Baseline	-0.477	-0.598	22.8%	49.8	-0.651	<0.001
CSQ	Decrease pain	Change	0.345	0.281	9.6%	23.8	0.184	<0.001
CSQ	Decrease pain	Baseline	-0.044	0.165	6.2%	16.7	0.131	<0.001
HADS	Depression	Change	0.278	0.180	3.9%	11.2	0.162	0.001
SF-36	Physical function	Change	0.254	0.247	3.4%	11.3	0.203	0.001
HADS	Depression	Baseline	0.043	0.166	3.4%	10.3	0.119	0.002
SF-36	Physical function	Baseline	0.039	0.283	2.0%	6.4	0.245	0.013
SF-36	Social functioning	Change	0.316	0.155	1.3%	4.7	0.078	0.032
Smoking			0.135	0.183	1.3%	4.4	4.335	0.038
Sports		Baseline	0.143	0.134	0.8%	2.9	1.358	0.090
**Functional improvement: NASS function** (explained variance: 53.3%)								
Constant							14.587	<0.001
CSQ	Catastrophizing	Change	0.440	0.261	19.4%	40.6	0.194	<0.001
NASS	Function	Baseline	-0.373	-0.537	11.4%	27.8	-0.482	<0.001
NASS	Pain	Change	0.378	0.390	11.3%	32.6	0.235	<0.001
NASS	Pain	Baseline	-0.084	0.264	5.9%	18.9	0.177	<0.001
HADS	Depression	Baseline	-0.078	0.215	2.0%	6.7	0.133	0.006
HADS	Depression	Change	0.440	0.279	1.9%	6.2	0.222	<0.001
Sex			0.181	0.174	1.5%	5.1	4.204	0.026
**Working capacity improvement** (explained variance: 61.6%)								
Constant							15.171	<0.001
Working capacity		Baseline	-0.753	-0.780	56.7%	221.0	-0.764	<0.001
NASS	Function	Baseline	0.028	0.210	2.5%	10.1	0.120	0.006
Age			-0.114	-0.180	1.8%	7.7	-0.134	0.020
Comorbidities			-0.109	-0.133	0.7%	3.0	-0.971	0.087

Legend: SF-36: Short Form 36. NASS: North American Spine Society cervical spine self-assessment instrument. CSQ: Coping Strategies Questionnaire. HADS: Hospital Anxiety and Depression Scale. Admission: baseline score, Change: Change of the score between baseline and follow-up.

**Table 2:** Multivariate regression of change in pain, function, and working capacity at the 6 month follow up (n = 103)

			**Bivariate**	**Multivariate**				
**Covariate**			**Correlation**	**Partial correlation**	**Explained variance**	**F change**	**Regression coefficient**	**Coefficient’s significance**
**Pain relief: NASS pain** (explained variance: 72.1%)								
Constant							-8.114	0.269
NASS	Pain	Baseline	-0.595	-0.734	35.5%	53.570	-0.860	<0.001
NASS	Function	Change	0.508	0.464	14.8%	28.776	0.615	<0.001
NASS	Function	Baseline	0.052	0.509	13.8%	36.854	0.665	<0.001
HADS	Anxiety	Baseline	-0.109	-0.311	2.6%	7.243	-0.257	0.008
Sports		Baseline	0.149	0.197	1.5%	4.692	2.510	0.033
Working capacity		Baseline	0.328	0.253	1.5%	4.608	0.200	0.034
CSQ	Catastrophizing	Change	0.348	0.260	1.5%	4.378	0.232	0.039
Smoking			-0.125	-0.193	1.1%	3.519	-5.684	0.064
**Functional improvement: SF-36 physical functioning** (explained variance: 63.4%)								
Constant							30.492	<0.001
HADS	Depression	Change	0.453	0.461	20.5%	24.976	0.361	<0.001
SF-36	Physical function	Baseline	-0.436	-0.689	19.3%	30.779	-0.622	<0.001
HADS	Depression	Baseline	0.028	0.368	12.2%	24.163	0.250	<0.001
SF-36	Bodily pain	Change	0.445	0.431	6.6%	14.929	0.303	<0.001
SF-36	Bodily pain	Baseline	-0.084	0.228	2.3%	5.356	0.202	0.023
Age			-0.148	-0.209	1.4%	3.475	-0.194	0.065
Sports		Baseline	-0.046	0.173	1.1%	2.822	1.756	0.096
**Working capacity improvement** (explained variance: 58.2%)								
Constant							-0.358	
Working capacity		Baseline	-0.526	-0.686	27.7%	37.166	-0.881	<0.001
NASS	Pain	Change	0.335	0.538	13.7%	22.493	0.400	<0.001
CSQ	Catastrophizing	Baseline	0.173	0.243	5.7%	10.142	0.186	0.002
Sports		Baseline	-0.054	-0.291	3.7%	7.848	-3.668	0.006
NASS	Pain	Baseline	-0.049	0.208	3.3%	6.195	0.186	0.015
SF-36	Physical function	Baseline	0.159	0.282	2.3%	4.600	0.227	0.035
Comorbidities			-0.063	-0.202	1.8%	3.863	-2.184	0.052

Legend: NASS: North American Spine Society cervical spine self-assessment instrument. CSQ: Coping Strategies Questionnaire. SF-36: Short Form 36. HADS: Hospital Anxiety and Depression Scale, Admission: baseline score, Change: Change of the score between baseline and follow-up.

Regression coefficients, their level of significance for prediction of the dependent variable, as well as bivariate (a priori, independent) and partial correlations (a posteriori, dependent from the other covariates in the model) were reported. Attrition bias, i.e. selection bias due to dropped-out patients, was assessed using logistic regression with completer/drop-out as the dependent variable and the key demographic and disease-relevant covariates as well as the covariates that were significantly associated in the models from baseline to discharge as independent variables [25,26]. If a covariate attained significance in those models, the corresponding linear model was corrected for attrition [25,26].

## Results

A total of 185 patients were included at baseline, while 10 participants dropped out between admission and discharge (4 patients because of premature discharge, 1 patient had a new accident, 1 because of illegal drug abuse and 4 patients because they refused further therapy). For assessment, 175 patients had complete data on discharge, and 103 at the 6-month follow-up. In between, 72 patients dropped out: 66 patients did not answer the follow-up questionnaires, 3 patients had had a new accident, 1 patient moved to an unknown address, 1 patient had family problems, and 1 patient was free of pain after face surgery. More detailed information about inclusion and exclusion data in the form of flow chart can be obtained from the afore-mentioned outcome study [13].

Sociodemographic and disease-relevant data at baseline are listed in Table 3. In summary, a typical patient might be described as “a slim, young woman, well educated and living in partnership”. The outcome measures are shown in Table 4. At all time points, the scores were significantly below the norm (all *p* < 0.001). Most of the effect sizes reflecting changes of health and abilities showed significant improvements.

**Table 3:** Sociodemographic and disease-relevant data at baseline (n = 175)

Female	79.4%
Living with partner/spouse	72.0%
Education	
Basic school (8-9 years)	7.6%
Vocational training	14.0%
College	52.3%
High school/university	26.1%
Smoker	36.3%
Sports	
None	
<1 hour/week	
1-2 hours/week	
>2 h/w	
Analgesic medication on admission	61.1%
Antidepressive medication on admission	25.7%
Comorbitities (n)	
None	16.0%
1	34.9%
2	29.7%
3	13.7%
4 or more	5.7%
Car accident	78.9%
Working capacity (hours/week)	
0-5	43.4%
6-10	5.7%
11-15	10.8%
16-20	9.7%
21-25	10.8%
26-30	6.8%
31-35	5.2%
36-40	3.5%
41-45	3.5%
46-50	0.6%
Age (years): mean (SD)	37.4 (11.7)
Disease duration (months): mean (SD)	13.3 (10.7)
Body mass index: mean (SD)	24.3 (4.7)

Legend: SD: Standard deviation.

**Table 4:** Measures and outcome during the course

			**Admission → discharge (n = 175)**			**Admission → 6 month follow-up (n = 103)**		
		**Norm mean**	**Mean (SD)**	**Mean (SD)**	**ES**	**Mean (SD)**	**Mean (SD)**	**ES**
**SF-36**	Physical functioning	87.3	55.8 (17.7)	67.0 (18.0)	0.63*	55.7 (18.3)	70.5 (18.9)	0.81*
	Bodily pain	62.9	21.0 (14.0)	29.5 (15.7)	0.61*	21.4 (14.3)	30.4 (20.9)	0.63*
	Social functioning	83.9	42.5 (26.1)	47.0 (25.7)	0.17*	45.3 (25.8)	50.7 (25.9)	0.21
**NASS**	Function	-	59.2 (15.2)	62.6 (16.5)	0.22*	59.8 (15.3)	66.1 (18.5)	0.41*
	Pain	-	17.5 (17.6)	28.3 (22.5)	0.61*	17.7 (19.1)	26.3 (21.2)	0.45*
**HADS**	Depression	81.6	62.3 (19.8)	67.3 (20.5)	0.25*	63.8 (20.4)	66.5 (22.1)	0.13
	Anxiety	77.8	61.9 (19.1)	62.8 (20.3)	0.05	63.3 (19.1)	66.2 (21.0)	0.15
**CSQ**	Catastrophizing	-	58.9 (18.7)	66.0 (20.5)	0.38*	60.1 (19.2)	66.4 (22.5)	0.33*
	Activity level	-	62.4 (15.4)	64.3 (15.0)	0.12	64.6 (14.6)	66.4 (14.7)	0.12
	Control pain	-	44.6 (20.0)	48.1 (20.6)	0.18*	45.0 (19.6)	50.5 (20.5)	0.28*
	Decrease pain	-	42.1 (18.5)	47.0 (19.7)	0.26*	43.2 (18.1)	46.0 (19.2)	0.15
**Work**	Working capacity (h/w)	-	13.3 (13.7)	18.9 (9.6)	0.40*	15.6 (14.2)	7.0 (19.4)	0.61*

Legend: SF-36: Short Form 36. NASS: North American Spine Society cervical spine self-assessment instrument. HADS: Hospital Anxiety and Depression Scale. CSQ: Coping Strategies Questionnaire. Norm: population based normative values. Mean: arithmetic mean. SD: Standard deviation. ES: Effect size. h/w: hours/week. *Significant effects (p < 0.050, Wilcoxon test).

At discharge from the rehabilitation program, all models showed high fit to the changes in the dependent variables attaining explained variances of between 53.3% and 61.6% (Table 1). Those using NASS pain (explained variance 47.6%) and SF-36 physical functioning (44.6%) were lower than those of the corresponding constructs using SF-36 bodily pain (54.6%) and NASS function (53.3%), and were therefore not listed. The most important associative factor for high pain relief (SF-36) was a low pain score at baseline (22.8% explained variance), followed by high improvement in CSQ decrease pain (9.6%), and low baseline score of CSQ decrease pain (6.2%), whereas high improvement in HADS depression and all other variables explained less than 5%. High functional improvement (NASS) was associated with high reduction of CSQ catastrophizing (19.4% explained variance), low baseline NASS function (11.4%), NASS pain relief (11.3%), and baseline NASS pain (5.9%). High improved working capacity was almost only dependent on low working capacity at baseline (56.7% explained variance).

At the 6 month follow-up (i.e. at home), all models showed high to very high fit to the changes in the dependent variables attaining explained variances of between 58.2% and 72.1% (Table 1). Again, the models of SF-36 bodily pain (explained variance 61.5%) and NASS function (59.0%) were not listed due to lower fit than NASS pain (72.1%) and SF-36 physical functioning (63.4%). The most important associative factor for high pain relief (NASS) was the NASS baseline pain (35.5%), high improvement in NASS function (14.8% explained variance), and low baseline score on NASS function (13.8%). The remaining variables explained the variance by less than 5%. High functional improvement (SF-36) was associated with high reduction of HADS depression (20.5% explained variance), low baseline SF-36 function (19.3%) and high baseline depression on the HADS (12.2%), as well as serious baseline pain on the SF-36 (6.6%). High improved working capacity was associated with low baseline working capacity (27.7% explained variance), NASS pain relief (13.7%), and low baseline CSQ catastrophizing (5.7%).

Attrition bias analysis detected no significant covariates that predicted whether a participant would be a completer or drop-out (between discharge and the 6 month follow-up) for pain and function at the 6 month follow-up. For change of working capacity, baseline working capacity was unequally distributed between the completers and the drop-outs (p = 0.011) and correction for attrition bias was performed for that model.

## Discussion

This prospective cohort study investigated associative factors for pain relief, better physical function, and improved working capacity after inpatient rehabilitation of WAD. The achieved multivariate linear models attained very good fit explaining variances between 53.3% and 72.1%. At baseline, health and quality of life of the subjects were significantly worse than expected compared to the general population [13]. Patients reported small to moderate improvements at discharge from the 4 week inpatient pain program (ES of the three dependent variables 0.40-0.63), and 5 months later at home (ES 0.61-0.81).

Improvement was positively associated with a low baseline value for each of the three dimensions at both discharge and the 6 month follow-up (explained variances 11.4%-56.7%), meaning that persons having much pain and high disability reported higher pain relief and functional improvements at the follow-up than those with less pain and better function. The second most predictive independent variables were dimensions of affective health and pain coping. Relief of depression and low baseline depression were highly associated with improved physical function (especially at the 6 month follow-up: 20.5% explained variance) and lower with pain relief (at discharge: 3.9% explained variance). The CSQ single item, i.e. asking about the self-perceived ability to decrease pain, was an important predictor for pain relief (at discharge: 6.2 and 9.6% explained variance). Low baseline catastrophizing, and reduction of catastrophizing were associated with improvements in all three dependent variables. For improved function at discharge, reduction of catastrophizing was the most important predictor (explained variance 19.4%). These findings confirmed the main hypothesis of the study. Low baseline pain and relief of pain (up to 11.3% explained variance) were associated with improvement of function and vice versa (up to 14.8% explained variance).

This study design does not allow for causal conclusions. However, daily clinical experience suggests that pain relief, improved physical function, and working capacity are circularly associated to each other and, therefore, there may be a partial causal component since there is low to moderate evidence that specific stretching and strengthening exercise relieve chronic neck pain [27]. Active physiotherapy, functional stability and mobility of the cervical spine may ameliorate pain in many patients but not in all. Our data suggest that patients suffering from severe pain and/or severe disability were more likely to improve and to profit from rehabilitation, because low baseline levels of pain and function were most associated with improvement in these dimensions. Physical therapy and psychotherapy were delivered during the stay and organized for continuation subsequent to discharge. Information about the disease and education on how to continue home exercises after discharge and how to transfer the newly acquired knowledge to daily life seemed to maintain improvements as was observed between discharge from rehabilitation and the 6 month follow-up at home [28,29].

During the inpatient program, patients were confronted with their lack of coping, and they learned to improve coping strategies. After the pain program, many participants were treated by psychiatrists for depression, anxiety, and coping/catastrophizing due to proven and postulated mechanisms [11,30-36]. Psychological factors were found to have more relevance for recovery than collision severity with regard to prediction of duration and severity of WAD [10]. Recovery and better health were associated with lower levels of pain catastrophizing, rumination, magnification, and helplessness three months or later after trauma [11]. Catastrophizing was linked to heightened emotional distress and disability, as well as a more intense pain experience, and more pronounced displays of pain behavior [7,32-34]. In the early stages of WAD, fear of movement was a predictor of next day pain and disability, which was significantly associated with chronic persistence of pain [28]. The hypothesized vicious circle of more pain and less function may partly be broken by effectively modifying coping factors and depression [7]. According to the fear and avoidance model, the results underpin the concept that pain relief can be enhanced by improvement of upper cervical function (operant behavioral therapy), and increased ability to decrease pain by learning coping strategies [7,37-39].

A strength of our study is its observational and “naturalistic” design without an artificial process of patient selection, allocation, or adaptation of the intervention. The study design aimed to be close to daily therapeutic and clinical reality. To measure clinical symptoms, we used standardized questionnaires which assess parameters in all dimensions of the WHO concept for International Classification of Function (ICF), like 1) Function (function/impairment of tissue or organ system), 2) Disability (functional impairment of the whole person) and 3) Health (participation/restriction in social interaction) [40]. The SF-36 measured in all three dimensions, HADS in dimension two, and the CSQ in dimensions two and three. In order to achieve a holistic bio-psycho-social approach we combined the questionnaires to cover all dimensions. The self-assessments used were validated and standardized especially for whiplash disorders [41]. Attrition bias was assessed and the model for change of working capacity at the 6 month follow-up was corrected for attrition bias. All models fulfilled the rule that at least 10 observations (i.e. patients) per included covariate have to be available for the regression to avoid overestimated and underestimated variances [42].

Limitations of the study are the high drop-out rate after discharge leading to possible attrition bias. Furthermore, the design was non-randomized and not controlled, which is acceptable for an association study. Data modeling was based on linear regression on the assumption that characteristics are linearly linked. A complex polynomial approximation would be more accurate for calculation. However, in most polynomial models, the linear term is by far the most predictive term compared to quadratic and cubic terms. Linear regression makes results clinically easier to interpret. Although many findings of the study correspond to data reported in the existing literature, generalizability of the results should be limited to inpatient rehabilitation and the first phase thereafter.

## Conclusion

Pain relief, improved physical function and working capacity were circularly associated with each other. This empirical finding supports the existence of a corresponding hypothetical circle as postulated by previous studies, clinical experience and intuition. Coping (catastrophizing and ability to decrease pain) and depression may act as important effect modifiers in this circle. These findings offer toe-holds for optimized therapy of chronic WAD.

## Abbreviations

BMI: Body mass index; CSQ: Coping strategies qestionnaire; HADS: Hospital Anxiety and Depression Scale; ICF: International Classification of Function; MTT: Medical training therapy; NASS: North American Spine Society; QTF: Quebec Task Force; SF-36: Short form 36; WAD: Whiplash associated disorders; WHO: World Health Organisation; ZIHKo: Zurzacher Interdisziplinäres HWS Konzept, (Zurzach Interdisciplinary Cervical Spine Concept).

## Competing interests

All authors report no competing interests with regard to this study.

## Authors’ contributions

All authors have made substantial contributions to conception or design of the study, acquisition, analysis and/or interpretation of data; FH, FA, AA and ARG have been mainly involved in drafting the manuscript or revising it critically for important intellectual content; and all authors have given final approval of the version to be published.

## Pre-publication history

The pre-publication history for this paper can be accessed here:

http://www.biomedcentral.com/1471-2474/15/130/prepub
