# Correction: multidimensional associative factors for improvement in pain, function, and working capacity after rehabilitation of whiplash associated disorder: a prognostic, prospective outcome study

**DOI:** 10.1186/1471-2474-15-195

**Published:** 2014-06-20

**Authors:** Felix Angst, Andreas R Gantenbein, Susanne Lehmann, Françoise Gysi-Klaus, André Aeschlimann, Beat A Michel, Frank Hegemann

**Affiliations:** 1 Rehabilitation Clinic "RehaClinic", Bad Zurzach, Switzerland; 2 Department of Rheumatology, Physical Medicine and Rehabilitation, University Hospital of Zurich, Zurich, Switzerland

## Correction

After publication of this work [1], we became aware of some typing errors, missing data and ambiguities in the results and discussion.

1) In the results, second paragraph, second last sentence, it has to be clarified: High functional improvement (NASS) was associated with high reduction of CSQ catastrophizing (19.4% explained variance), low baseline NASS function (11.4%), NASS pain relief (11.3%), and low baseline NASS pain (5.9%).

2) In the results, third paragraph, the results of the 6 month follow-up rely on Table two.

3) In the same paragraph later on, the following is more precise: The most important associative factor for high pain relief (NASS) was a low NASS baseline pain level (reflecting high pain) (35.5%), high improvement in NASS function (14.8% explained variance), and a low baseline score on NASS function (13.8%). And later on:

High functional improvement (SF-36) was associated with high reduction of HADS depression (20.5% explained variance), low baseline SF-36 function (19.3%) and high baseline depression on the HADS (12.2%), as well as pain relief on the SF-36 (6.6%).

4) In Table three (Table 1 here), missing data of the category sports have been added, see below.

**Table 1:** Sociodemographic and disease-relevant data at baseline (n = 175)

	
Female	79.4%
Living with partner / spouse	72.0%
Education	
Basic school (8–9 years)	7.6%
Vocational training	14.0%
College	52.3%
High school / university	26.1%
Smoker	36.3%
Sports	
None	33.7%
<1 hour/week	24.4%
1–2 hours/week	18.6%
>2 hours/week	23.3%
Analgesic medication on admission	61.1%
Antidepressive medication on admission	25.7%
Comorbitities (n)	
None	16.0%
1	34.9%
2	29.7%
3	13.7%
4 or more	5.7%
Car accident	78.9%
Working capacity (hours/week)	
0-5	43.4%
6-10	5.7%
11-15	10.8%
16-20	9.7%
21-25	10.8%
26-30	6.8%
31-35	5.2%
36-40	3.5%
41-45	3.5%
46-50	0.6%
Age (years): mean (SD)	37.4 (11.7)
Disease duration (months): mean (SD)	13.3 (10.7)
Body mass index: mean (SD)	24.3 (4.7)

Legend: SD: Standard deviation.

5) In the discussion, third paragraph, the following has to be clarified: Our data suggest that patients suffering from severe pain and/or severe disability were more likely to improve and to profit from rehabilitation, because low baseline levels of the pain scores (reflecting much pain) and of the function scores (reflection much disability) were most associated with improvements in these dimensions.

These corrections substantially improve comprehensibility and distinctness of the data and the interpretations. However, the corrections do not alter the results and the conclusions of the study.

## Pre-publication history

The pre-publication history for this paper can be accessed here:

http://www.biomedcentral.com/1471-2474/15/195/prepub
